# Detection of cryptic diversity in lizards (Squamata) from two Biosphere Reserves in Mesoamerica

**DOI:** 10.3897/CompCytogen.v14i4.57765

**Published:** 2020-12-22

**Authors:** Riccardo Castiglia, Oscar Alberto Flores-Villela, Alexandra M. R. Bezerra, Ekaterina Gornung, Flavia Annesi, Luis Antonio Muñoz-Alonso, Emanuela Solano

**Affiliations:** 1 Dipartimento di Biologia e Biotecnologie ‘Charles Darwin’, Università di Roma ‘La Sapienza’, via A. Borelli 50, CAP 00151, Rome, Italy Università di Roma ‘La Sapienza’ Rome Italy; 2 Museo de Zoologia Fac. de Ciencias, Universidad Nacional Autónoma de México, A.P. 70-399, Mexico D.F. 04510, Mexico Universidad Nacional Autónoma de México Mexico Mexico; 3 Mastozoologia/COZOO, Museu Paraense Emilio Goeldi, Campus de Pesquisa, Av. Perimetral 1901, CEP 66077-830, Belém, PA, Brazil Museu Paraense Emilio Goeldi Belém Brazil; 4 El Colegio de la Frontera Sur. Conservación de las Biodiversidad. Carretera Panamericana y Periférico Sur s/n. C.P. 29290, San Cristóbal de las Casas, Chiapas, México El Colegio de la Frontera Sur. Conservación de las Biodiversidad San Cristóbal de las Casas Mexico

**Keywords:** Cytotaxomy, DNA, herpetofauna, taxonomy

## Abstract

A combined approach based on karyology and DNA taxonomy allowed us to characterize the taxonomic peculiarities in 10 Mesoamerican lizard species, belonging to six genera and five families, inhabiting two Biosphere Reserve in Chiapas, Mexico: La Sepultura Biosphere Reserve, and Montes Azules Biosphere. The karyotypes of four species, *Phyllodactylus* sp. 3 (*P. tuberculosus* species group) (2n = 38), *Holcosus festivus* (Lichtenstein et von Martens, 1856) (2n = 50), *Anolis lemurinus* Cope, 1861 (2n = 40), and *A. uniformis* Cope, 1885 (2n = 29–30) are described for the first time, the last one showing a particular X_1_X_1_X_2_X_2_/X_1_X_2_Y condition. In *Aspidoscelis deppii* (Wiegmann, 1834) (2n = 50) and *Anolis capito* Peters, 1863 (2n = 42), we found a different karyotype from the ones previously reported for these species. Moreover, in *A. capito*, the cytogenetic observation is concurrent with a considerable genetic divergence (9%) at the studied mtDNA marker (MT-ND2), which is indicative of a putative new cryptic species. The skink *Scincella cherriei* (Cope, 1893), showed high values of genetic divergence (5.2% at 16S gene) between the specimens from Montes Azules and those from Costa Rica and Nicaragua, comparable to the values typical of sister species in skinks. A lower level of genetic divergence, compatible with an intraspecific phylogeographic structure, has been identified in *Lepidophyma flavimaculatum* Duméril, 1851. These new data identify taxa that urgently require more in-depth taxonomic studies especially in these areas where habitat alteration is proceeding at an alarming rate.

## Introduction

The Mesoamerican biota, with its number of endemics in different groups of taxa is one of the most diverse and interesting on the planet (for revision see Ríos-Muñoz 2013). The herpetofauna of this region is one of the richest in taxa groups in the continent (Savage 1982; Wilson and Johnson 2010). Part of this richness is managed and protected under the Biosphere Reserves (UNESCO 2018), which comprises terrestrial, marine, and coastal ecosystems and promote conservation of biodiversity along with its sustainable use. In Mexico, 42 Biosphere Reserves have been created since 1977, encompassing the majority of the environments found in the country (Udvardi 1984).

Saurians are one of the most representative group in terms of karyotypic diversification among reptiles (Olmo and Signorino 2005) and the study of chromosomal evolution in reptiles has received much attention thanks to advanced molecular cytogenetics tools (Deakin and Ezaz 2019; Rovatsos et al. 2019). However, even conventional karyotypes data can be informative in taxonomy (e.g. Santos et al. 2007; Matos et al. 2016; Hardy et al. 2017; Giovannotti et al. 2017).

Our previous studies aimed to genetically characterize the lizard community of a tropical dry forest in the Chamela-Cuixmala Biosphere Reserve (Jalisco state, Mexico) by means of DNA and chromosome analysis (Castiglia et al. 2009, 2010). Even if the herpetofauna of the area was previously quite well known, with two field guides already published (García and Ceballos 1994; Ramirez-Bautista 1994), several new karyotypes of unstudied species were described and species that showed high intraspecific genetic divergence were identified. Later, these findings were confirmed by more extended studies and led to the description of new species (García-Vázquez et al. 2018a, b; Ramírez-Reyes and Flores-Villela 2018).

This study aims to extend the genetic characterization of lizard species in two additional Biosphere Reserves in Mesoamerica: La Sepultura and Montes Azules Biosphere Reserves, both in Chiapas state, Mexico. From a biotic perspective, Chiapas is an area of transition between the herpetofauna of Mexico and that of Central America, along with the one of the Yucatan Peninsula (Lee 1996). Its herpetofauna, is the second largest among all the states in Mexico. The level of endemism is also high with 17.6% of species limited to Mexico. However, habitat alteration in Chiapas is proceeding at a rapid rate, as a result of rising human population growth and the damage that this creates to natural systems (Johnson et al. 2015).

In the present study, karyotypes of the sampled species have been characterized. Then, in conjunction with karyotype data, mtDNA genes for different species, sequenced here and available from GeneBank, were used as molecular markers to identify new putative cryptic species and/or new evolutionarily significant units (ESU) (Funk and Fa 2006).

## Material and methods

### Study area and sampling specimens

**Figure 1.**
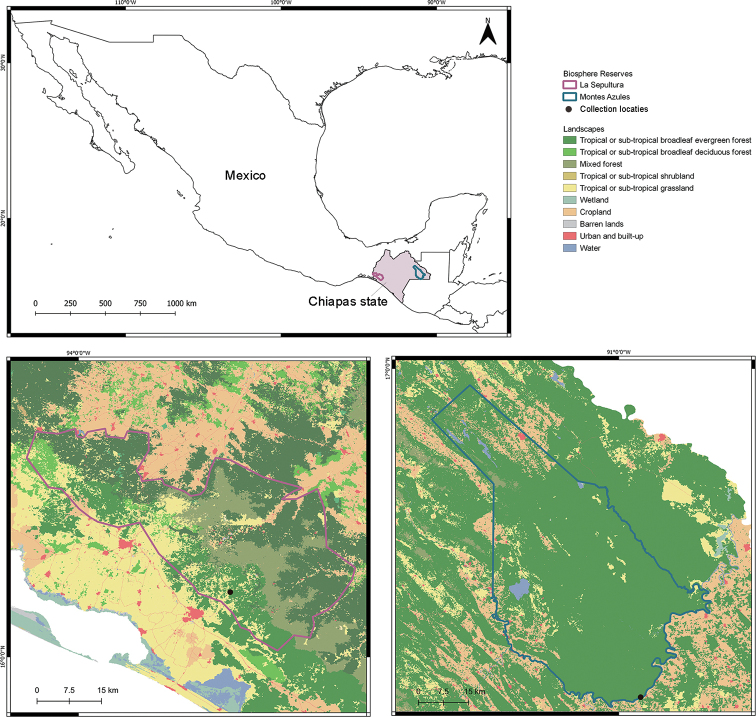
Map showing the collection localities of specimens used in this study, in La Sepultura and Montes Azules Biosphere Reserves, Chiapas state, Mexico.

Lizard specimens here analyzed were sampled in two localities: La Sepultura Biosphere Reserve, during September 2009, and Montes Azules Biosphere Reserve during 2012, Chiapas state, Mexico (Fig. 1), hereafter La Sepultura and Montes Azules, respectively. The physiographic profile of Chiapas state consists of a set of layered regions oriented in a NW–SE direction. The sampled areas belong to two different physiographic regions, respectively: La Sepultura belongs to the Pacific Coastal Plain and is characterized mainly by dry tropical forest in its lower parts, while Montes Azules belongs to the Eastern Highlands with the evergreen tropical forest (García de Miranda and Falcón de Gyves 1986). Maps were generated in QGIS version 2.18.9 ‘Las Palmas’ (QGIS 2017), using map shapes from North American Land Change Monitoring System (NALCMS 2020) for North American ecosystems and CONANP (2019) for protected and conservation areas of Mexico.

The specimens were captured by hand in active searching in random walks along the surveyed localities. Details on voucher numbers, genes sequenced, chromosome complements and sampled localities, for each species are shown in Table 1. Taxonomic classification and species distribution follow Uetz et al. (2020). All the tissues and chromosomal samples were labeled with RCMX (field numbers of Riccardo Castiglia) and housed in the herpetological collection of the Museum of Comparative Anatomy of Vertebrates “Battista Grassi” of the University “La Sapienza”, Rome, Italy. The voucher specimens, preserved in 80% ethanol, were partly kept in the Museum of Zoology “Alfonso L. Herrera”, Mexico City, D.F. (OFV field number of Oscar Flores-Villela), and the remaining specimens in the Museum of Comparative Anatomy of Vertebrates “Battista Grassi”.

### Karyotype and molecular analysis

For karyotyping, specimens were injected with a 1:1000 solution of Velbe (Lilly) for one hour. The femurs, vertebral column, and testes were removed, crushed and left in hypotonic solution (0.075 M KCl) for 40 minutes at room temperature. Cells were collected by centrifugation and fixed with a methanol-acetic acid solution (3:1). Metaphase plates were prepared by standard air-drying method and slides were stained with Giemsa (pH = 7). Metaphases images were captured with a Photometrics Sensys 1600 digital camera (Roper Scientific Photometrics, Tucson, AZ). For each species, we identified the diploid number (2n), the number of macro- and microchromosomes, and the morphology of macrochromosomes. In some species, it was also possible to assess the morphology of the largest microchromosomes.

For molecular analyses, tissues were extracted from liver and body muscle, and preserved in 100% ethanol. A fragment of the mtDNA genome was sequenced for each species, and the sequenced genes were either cytochrome b (MT-CYB), NADH-ubiquinone oxidoreductase core subunit 2 (MT-ND2) or mitochondrially encoded 16S rRNA (16S) (Table 1). The choice of molecular markers depended primarily on the availability of DNA sequences of congeneric and/or conspecific specimens in the GenBank (see results section for accession numbers of sequences downloaded from GenBank).

The QIAmp tissue extraction kit (Qiagen) was used for DNA extraction. We used the universal primers L14841 and H15149 (Kocher et al. 1989) for MT-CYB amplification and two pairs of primers, L4160-ND1 / H4980-ND2 and L4437 tRNAMet / H5934a COI, designed by Macey et al. (1999) for the MT-ND2 gene. Sequences of 16S gene were obtained using the primers 16SA-L and 16SB-H described in Palumbi et al. (1991). The standard PCR procedure was applied as detailed in Castiglia et al. (2010).

Molecular identification of the specimen was performed with the BLAST algorithm (https://blast.ncbi.nlm.nih.gov/Blast.cgi) using, the newly obtained sequences and searching for highly similar sequences (Mega BLAST) on the entire nucleotide collection database. When sequence identity was below 98% the sequences were aligned with the sequences from the same species and/or same genus downloaded from GenBank. Phylogenetic relationships were evaluated with Bayesian inference (BI) and the BI tree was built with the software MrBayes v3.2.1 (Ronquist and Huelsenbeck 2003), under the assumption of a GTR + I + G (General Time Reversible) model of sequence evolution. The appropriate evolution model was chosen using the software jModeltest 2.1 (Darriba et al. 2012) following the Bayesian (BIC) and Akaike (AIC) information criteria. Two independent Markov Chain Monte Carlo (MCMC) analyses were run with four chains and two million generations sampling the chains every 1,000 generations. A burn-in of 10% of generated trees was applied. The software Tracer 1.7 (Rambaut et al. 2018) was used to check parameters convergence. Only the values of posterior probabilities (p.p.) major than 50 are reported on the tree. All the twenty-five new sequences are submitted to GenBank (Table 1).

For some species a TCS Parsimony Network (Clement et al. 2002) connecting haplotypes was obtained with popART (Leigh and Bryant 2015) to visualize mutational steps among main lineages. Gene abbreviation follows HUGO Gene Nomenclature Committee at the European Bioinformatics Institute (HGNC 2019).

## Results and discussion

**Figure 2.**
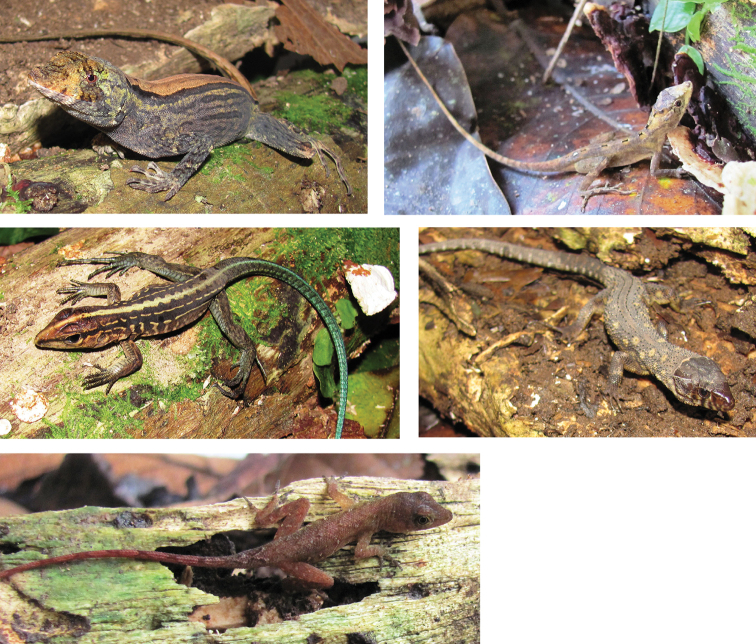
Some lizard species analyzed in present study (Photos by Riccardo Castiglia) **A***Anolis capito***B***Anolis lemurinus***C***Holcosus festivus***D***Lepidophyma flavimaculatum***E***Anolis uniformis*.

We obtained karyological and molecular data for 10 species (Fig. 2, Table 1), belonging to six genera and five families. The accounts below describe the species of lizards studied, with comments on their distribution, karyotypes, systematics, and voucher specimens. Voucher specimens with an asterisk (*) were karyotyped.

**Table 1.** Details of gene sequenced, chromosome complement and sampled localities, for each species studied in this work. In voucher numbers, OFV indicated those specimens held in the Museum of Zoology “Alfonso L. Herrera”, Mexico City; every other specimen is held in the Museum of Comparative Anatomy of Vertebrates “Battista Grassi” of the Rome University “La Sapienza”, Rome, Italy.

Taxon	Voucher	Gene sequenced	GenBank accession numbers	Karyotype	Locality
Squamata					
Scincidae					
*Scincella assata*	RCMX 85	16S	–	2n = 28 (7M + 14 m)	La Sepultura Biosphere Reserve
	RCMX 86		–		
	RCMX 92		MW265933		
*Scincella cherriei*	RCMX 219 (OFV 1197)	16S	MW265931	2n = 30 (7M + 16 m)	Montes Azules Biosphere Reserve
	RCMX 235		MW265932		
Phyllodactylidae					
*Phyllodactylus* sp.3	RCMX 67 RCMX 69 RCMX 93	MT-CYB	MW275909 MW275910 MW275911	2n = 38	La Sepultura Biosphere Reserve
Xantusiidae					
*Lepidophyma flavimaculatum*	RCMX 207 (OFV 1177)	MT-CYB	–	2n = 38 (18M + 20m)	Montes Azules Biosphere Reserve
	RCMX 208 (OFV 1178)		–		
	RCMX 212 (OFV 1179)		MW275912		
	RCMX 213 (OFV 1180)		MW275913		
	RCMX 232 (OFV 1255)		MW275914		
Teiidae					
*Aspidoscelis deppii*	RCMX 76	MT-CYB	MW275915	2n = 52 (28M + 24m)	La Sepultura Biosphere Reserve
*Holcosus festivus*	RCMX 223 (OFV 1213)	MT-ND2	MW275916	2n = 50 (26M + 24m)	Montes Azules Biosphere Reserve
	RCMX 224 (OFV 1214)		–		
	RCMX 233		MW275917		
*Holcosus undulatus*	RCMX 77	MT-ND2	MW275918	2n = 50 (26M + 24m)	La Sepultura Biosphere Reserve
Dactyloidae					
*Anolis capito*	RCMX 217 (OFV 1203)	MT-ND2	MW275927	2n = 42 (24M + 18m)	Montes Azules Biosphere Reserve
	RCMX 218 (OFV 1204)		MW275928		
*Anolis lemurinus*	RCMX 214 (OFV1186)	MT-ND2	MW275930	2n = 40 (24M + 16m)	Montes Azules Biosphere Reserve
	RCMX 225 (OFV 1215)		MW275929		
*Anolis uniformis*	RCMX 201 (OFV 1160)	MT-ND2	MW275919	2n = 29/30 (14M + 15/16m)	Montes Azules Biosphere Reserve
	RCMX 203		MW275925		
	RCMX 205 (OFV 1164)		MW275926		
	RCMX 206 (OFV 1176)		MW275920		
	RCMX 209 (OFV 1183)		MW275921		
	RCMX 210 (OFV 1173)		MW275922		
	RCMX 215 (OFV 1182)		MW275923		
	RCMX 226 (OFV 1211)		MW275924		

### Order Squamata

#### Family Scincidae

##### Genus *Scincella* Mittleman, 1950

The Mexican herpetofauna includes seven *Scincella* species that formerly belonged to the genus *Sphenomorphus* Fitzinger, 1843. They were reassigned to *Scincella* based on molecular phylogenetic analyses (Honda et al. 2003; Linkem et al. 2011). The two species, *Scincella assata* (Cope, 1864) and *S. cherriei* (Cope, 1893), belong to this group and are sister species following Linkem et al. (2011). Both of them have already been karyotyped in a recent study (Castiglia et al. 2013a, see comments below).

###### Scincella assata

Taxon classificationAnimaliaSquamataScincidae

(Cope, 1864)

69EED207-E760-54AA-8810-DA49A4F6EE96

####### Distribution.

This species is distributed from Colima state, Mexico, southwards to Chiapas state, on the Pacific coast, and towards the southwest to Guatemala and Honduras.

####### Samples.

RCMX85 (male*), RCMX86 (female*) and RCMX92 (female*) from La Sepultura, Chiapas, Mexico.

####### DNA taxonomy.

See below under *S. cherriei* (Cope, 1893) account.

####### Chromosomes.

The karyotype, described in Castiglia et al. (2013a) shows a diploid number of 2n = 28 and heteromorphic sex chromosomes. The diploid complement present four pairs of large metacentrics, two pairs of medium sized metacentrics, and one pair of heteromorphic (XY) sex chromosomes (pair 7; one small subtelocentric and one microchromosome). The remaining chromosomes are microchromosomes.

###### Scincella cherriei

Taxon classificationAnimaliaSquamataScincidae

(Cope, 1893)

E4F6BA83-076F-5F0F-8892-DFF8EF66EE97

####### Distribution.

This species inhabits Mexico, from central Veracruz to extreme southeastern Puebla, northern Oaxaca state, southwards to Central America on the Atlantic coast, including the Yucatan Peninsula in México, reaching the eastern Panama.

####### Samples.

RCMX219 (male) and RCMX235 (male*) from Estación Chajul, Selva Lacandona, Montes Azules, Chiapas state, Mexico.

####### DNA taxonomy.

**Figure 3.**
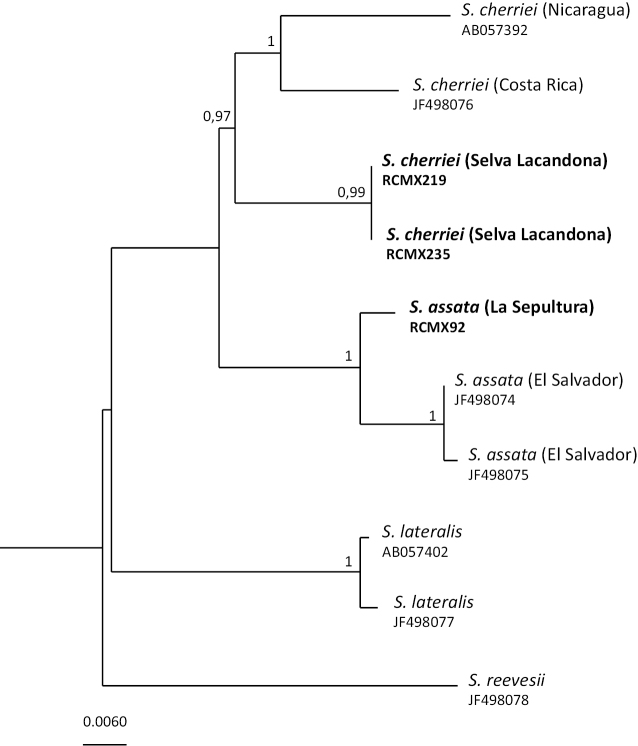
Bayesian phylogenetic tree (16S) of 16S haplotypes from Mexican *Scincella* species. In bold, the new individuals from this study; the geographic provenience of each individual is reported in brackets.

The BI phylogenetic tree has been performed on 448-bp alignment of the 16S gene for four individuals of *Scincella cherriei* [RCMX219 and RCMX235 from the Montes Azules, one from Costa Rica (JF498076) and one from Nicaragua (AB057392)] and three individuals of *Scincella assata* [RCMX92 from La Sepultura, and two from El Salvador (JF498074 and JF498075)]. *Scincella lateralis* (Say, 1822) (AB057402 and JF498077) and *S. reevesii* (Gray, 1838) (JF498078) were used as outgroups. The tree (Fig. 3) shows *S. assata* as a monophyletic and well supported group (p.p.: 1.0), including the individual from La Sepultura. The two individuals of *S. cherriei* from the Montes Azules, southern Mexico, form a well-supported group separated from the other two individuals from Costa Rica and Nicaragua that fall in a well distinct clade (p.p.: 1.0).

The genetic divergence between the two specimens of *S. cherriei* from the Montes Azules and *S. cherriei* from other localities is high (5.2%), comparable to the divergence between *S. assata* and *S. cherriei* (6.6%-6.2%). The nominal subspecies *S. c. cherriei* (Cope, 1893), was described from Palmar, Costa Rica, which is far from from the Montes Azules. The lineage of *S. cherriei* from the Montes Azules may represent a different taxon worthy of additional detailed morphological and genetic studies.

####### Chromosomes.

The karyotype, described in Castiglia et al. (2013a), shows a diploid number of 2n = 30 and in this case the presence of heteromorphic (XY) sex chromosomes. The diploid complement of *S. cherriei* differs from its sister species *S. assata* by the presence of an additional pair of microchromosomes.

#### Family Phyllodactylidae

##### Genus *Phyllodactylus* Gray, 1828

The genus *Phyllodactylus* is now constrained to the New World (Bauer et al. 1997; Gamble et al. 2008). Albeit there are more than 50 species in the genus, karyological data are very scant (Weiss and Hedges 2007; Blair et al. 2009; Nielsen et al. 2019). Recently, many species groups within the genus have been studied using molecular phylogenetic and species delimitation methods, and several additional cryptic species have been revealed (Blair et al. 2015; Koch et al. 2016; Ramírez-Reyes et al. 2017).

###### Phyllodactylus

Taxon classificationAnimaliaSquamataPhyllodactylidae

sp. 3 (P. tuberculosus species group, lineage A11 sensu Blair et al. 2015)

57E57C57-EE35-5FA8-8944-30EBCE22BC73

####### Distribution.

provisional distribution of this lineage, probably representing an undescribed species, is restricted to Pacific coast of eastern Oaxaca and western Chiapas states, Mexico (Blair et al. 2015).

####### Samples.

RCMX67 (female*), RCMX69 (male*) and RCMX93 (female*) from La Sepultura, Chiapas state, Mexico.

####### DNA taxonomy.

**Figure 4.**
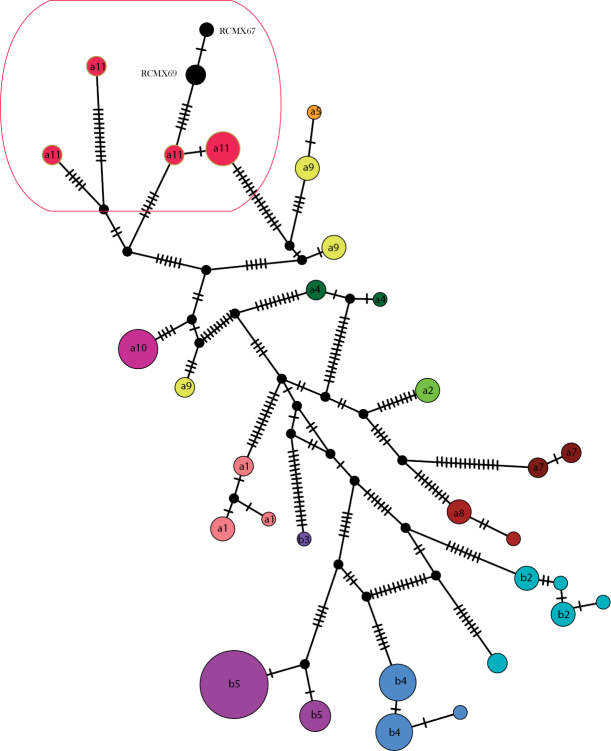
TCS network of MT-CYTB haplotypes of *Phyllodactylus tuberculosus* species group. The colors refer to the 11 lineages reported by Blair et al. (2015) for this species complex. The lineage “A11” and the new specimens here analysed are indicated (see text for further explanation).

Blair et al. (2015) reported the most complete phylogeny of the *Phyllodactylus tuberculosus* species group, defining the presence of 11 distinct lineages that represent separated species. We aligned the obtained 579-bp MT-CYB sequences from our samples to the 115 MT-CYB sequences of the 11 lineages reported by Blair et al. (2015) using *Tarentola mauritanica* (Linnaeus, 1758) (JQ425060) as the outgroup. The TCS network (Fig. 4) indicated that the haplotypes of our samples are similar those belonging to the lineage A11 (Blair et al. 2015), from Oaxaca and Chiapas states, and show a shallow genetic divergence (1.2%) compared to A11. Therefore, we provisionally assigned the samples from La Sepultura to this lineage.

####### Chromosomes.

**Figure 5.**
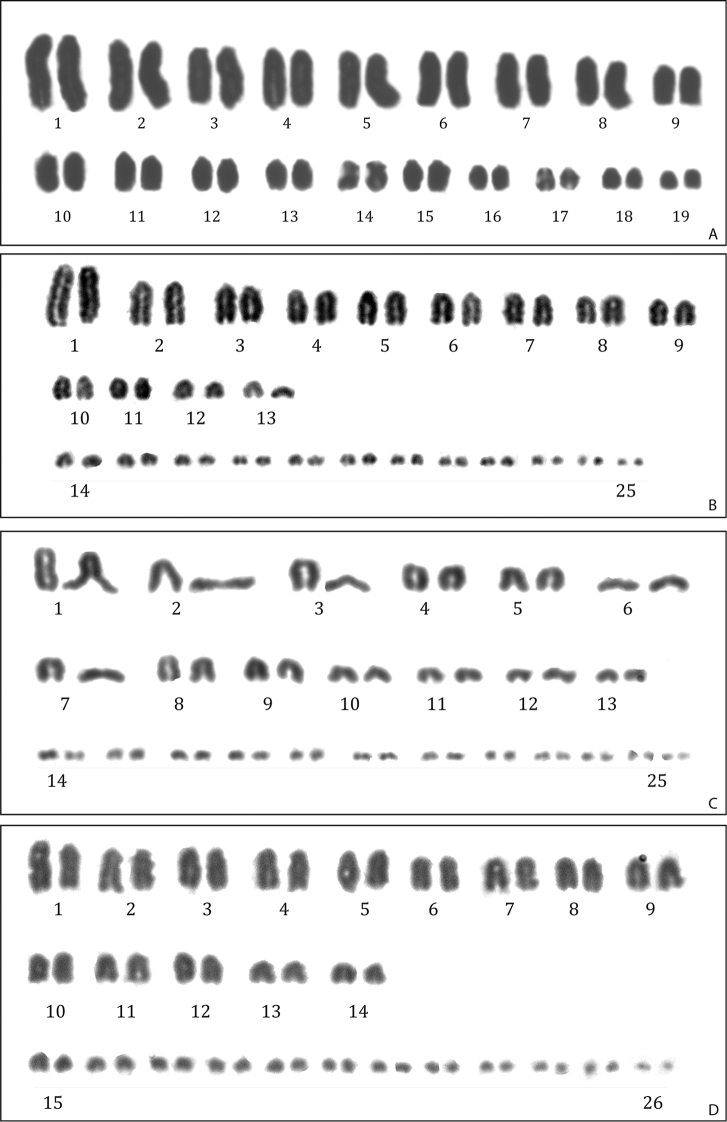
Karyotypes of **A***Phyllodactylus* sp.3 (2n = 38, RCMX69 male) **B***Holcosus festivus* (2n = 50, RCMX224 female) **C***Holcosus undulatus parvus* (2n = 50, RCMX77 female) **D***Aspidoscelis deppii* (2n = 52, RCMX76 female).

The first description of the karyotype of one species of the *P. tuberculosus* complex is reported here (Fig. 5A). The three specimens analyzed (two females and one male) showed a 2n = 38 with no distinction in macro- and microchromosomes. All chromosomes are telocentric with exception of two pairs of small metacentric chromosomes (pair 14). We found no evidence of heteromorphic sex chromosomes.

As previously reported, 2n = 38 is the most common karyotype found in species of the genus *Phyllodactylus* from the Pacific coast of Mexico (Castiglia et al. 2009; Murphy et al. 2009). Exceptions are constituted by *P. paucituberculatus* Dixon, 1960 and *P. lanei* Smith, 1935 (*sensu* Ramírex-Reyes and Flores-Villela 2018), which have 2n = 32 and 2n = 33–34, respectively (Castiglia et al. 2009). The 2n = 38 karyotype is normally all-acrocentric, except for some records in *P. bugastrolepis* Dixon, 1966 and *P. papenfussi* Murphy, Blair et Mendes de la Cruz, 2019 (Murphy et al. 2009). The ZW sex determination system has been found in *P. wirshingi* Kerster et Smith, 1955 (Nielsen et al. 2019) and, probably, in *P. lanei* (King, 1981). In all taxa, there is no distinct break between macro- and microchromosomes. The karyotype of the specimens from La Sepultura described here, is similar to the gekkonid karyotype defined by Gorman (1973). In fact, the typical gekkonid karyotype is composed of a series of acrocentric chromosomes, gradually decreasing in size, with few or no bi-armed chromosomes and no distinct boundary between macrochromosomes and microchromosomes (Bickham 1984). The 2n = 38 acrocentric karyotype is considered to be the ancestral in the families Gekkonidae, Diplodactylidae, and Eublepharidae. In Phyllodactylidae the chromosomal number ranges from 2n = 32 to 2n = 44 (Pellegrino et al. 2009). While the karyotype of the genus *Phyllodactylus* seems rather conservative, the pair of metacentric chromosomes in the here studied specimens indicates presence of intrachromosomal rearrangements (Pokorná et al. 2015). Therefore, these chromosomes may represent chromosomal markers for further investigation in this genus characterized by multiple cryptic species (Blair et al. 2015).

#### Family Xantusiidae

##### Genus *Lepidophyma* Duméril, 1851

The genus *Lepidophyma* comprises 20 recognized species and is particularly speciose in Mexico, where 15 species are endemic and, in some cases, restricted to a particular mountain landscape (Palacios-Aguilar et al. 2018). Only two species of this genus are widely distributed in Mexico and Central America: *L. smithii* Boucourt, 1876 and *L. flavimaculatum* Duméril, 1851. However, the former is paraphyletic with respect to *L. lineri* Smith, 1973 and the latter includes a previously unrecognized species from Chiapas state, Mexico (Noonan et al. 2013).

###### Lepidophyma flavimaculatum

Taxon classificationAnimaliaSquamataXantusiidae

Duméril, 1851

5FD2D565-49CA-520A-8893-A256103BB18E

####### Note.

Bezy and Camarillo (2002) did not recognize subspecies, although they admitted that populations of this taxon form a complex, therefore representing more than one taxon. It is the only vertebrate species with unisexual parthenogenetic populations that are of non-hybrid origin (Sinclair et al. 2010).

####### Distribution.

Found on the Gulf of Mexico coast from Veracruz and Oaxaca, crossing the base of the Yucatan peninsula, through Central America to Panama.

####### Samples.

RCMX207 (female*), RCMX208 (male*), RCMX212 (female*), RCMX213 (male*), and RCMX232 (female*) from Montes Azules, Chiapas state, Mexico.

####### DNA taxonomy.

**Figure 6.**
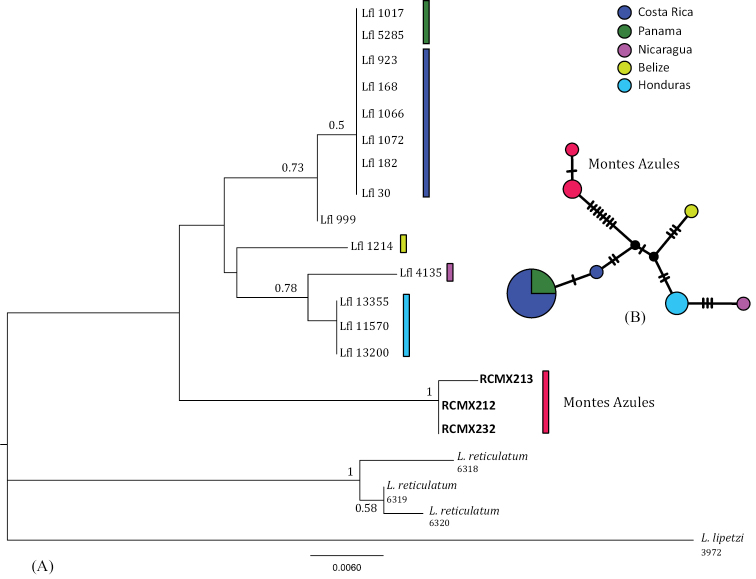
Bayesian phylogenetic tree (**A**) and TCS network (**B**) of 16S haplotypes belonging to *Lepidophyma flavimaculatum*. The colors refer to the geographic provenience of individuals. In bold, the new specimens from this study.

Our samples have been identified on a morphological basis as *Lepidophyma flavimaculatum*, a species already reported for Chiapas. We aligned our 309 bp MT-CYB sequences to the 14 haplotypes of the same species published in Sinclair et al. (2010) from Honduras, Nicaragua and Belize, as well as the unisexual populations from Costa Rica and Panama; *L. reticulatum* Taylor, 1955 and *L. lipetzi* Smith et Del Toro, 1977 were used as outgroups. The phylogenetic trees (Fig. 6A) showed that our samples are sister to the *L. flavimaculatum* clade, but it forms a separate and well supported lineage (p.p. = 1) with 3.9% of genetic divergence. The TCS network (Fig. 6B) confirms that the samples from Chiapas are differentiated from all the other populations of *L. flavimaculatum* by 8 substitutions, whereas the other haplotypes differ from each other by not more than 3 substitutions. The shallow distinction of the Chiapas population may reflect the phylogeographic structure of the species, in accordance with its distant geographical location. Moreover, Bezy (1989) found that Chiapas specimens are morphologically distinct from other southern Mexican samples. Therefore, additional comparative studies at the northern edge of the species range are needed.

####### Chromosomes.

**Figure 7.**
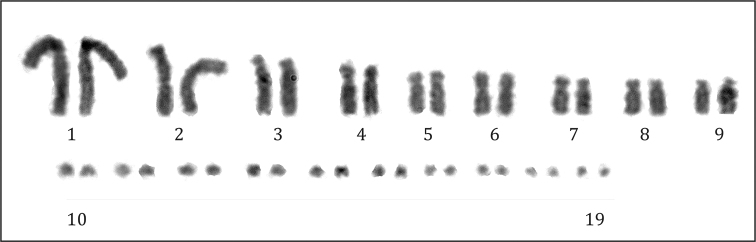
The karyotype of *Lepidophyma flavimaculatum* (2n = 38, RCMX208 male).

Diploid chromosome complements vary from 2n = 24 to 2n = 40 in Xantusiidae (Olmo and Signorino 2005). Within *Xantusia* Baird, 1859 the karyotypic formula is highly conserved with all studied species displaying 2n = 40, while the genus *Lepidophyma* is much more variable with diploid number ranging from 2n = 32 to 2n = 38 (Olmo and Signorino 2005). There is no evidence of heteromorphic sex chromosomes within the family, but recently a ZZ/ZW sex chromosomes system was described in the *X. henshawi* Stejneger, 1893 (Nielsen et al. 2020). In *L. flavimaculatum* unisexual parthenogenetic populations are known from Panama and southeastern Costa Rica, whereas northern populations are bisexual. All unisexual populations so far studied are diploid (2n = 38), except one mosaic individual (2n/3n) (Bezy 1972). All individuals presently analysed (Fig. 7) showed 2n = 38 with 18 macrochromosomes and 20 microchromosomes, as previously reported by Bezy (1972).

#### Family Teiidae

##### Genus *Aspidoscelis* Fitzinger, 1843

Species of the genus *Aspidoscelis* were previously included in *Cnemidophorus* Wagler, 1830, but based upon divergent morphological, molecular, and enzymatic characters the two genera were separated (Reeder et al. 2002). Thus, *Aspidoscelis* was resurrected for the North American *Cnemidophorus* clade containing 87 species included in the *A. deppii*, *A. sexlineata* and *A. tigris* species groups (and the unisexual taxa associated with them). *Aspidoscelis* occurs throughout most of North America (except Canada and much of northern United States), reaching the East and West Coasts of the United States, and ranging south through all Mexico and into Central America (Harvey et al. 2012).

The species groups differ also in their karyotypes. 2n = 52 is observed in the *deppii* group, 2n = 46 in the *sexlineata* group, and 2n = 46 with XY sex chromosomal system in the *tigris* group. Lowe et al. (1970) suggested a chromosomal evolution pattern through a reduction of the diploid number. This view has been slightly modified by Reeder et al. (2002), who considered that the ancestor probably had a karyotype of 2n = 50.

###### Aspidoscelis deppii

Taxon classificationAnimaliaSquamataTeiidae

(Wiegmann, 1834)

29B057AB-B74B-5E95-B291-DD8EF1196893

####### Distribution.

The species has a wide distribution from Morelos and Michoacan (Mexico) south to Guatemala, El Salvador, Honduras, Nicaragua and Costa Rica.

####### Samples.

RCMX76 (female*) from La Sepultura, Chiapas, Mexico.

####### DNA taxonomy.

The MT-CYB sequence (294-bp) is 4% divergent from GenBank sequences of *Aspidoscelis deppii* (KF555517-21) from Mexico (Playa Miramar, Tabasco). Despite the wide distribution, there are no studies on the intraspecific genetic variability of this species. It is a pity because this slight divergence in the MT-CYB could match with a different karyotype (see below).

####### Chromosomes.

In the genus *Aspidoscelis* chromosomal number ranges from 2n = 44 to 2n = 56, with some species showing triploid numbers, such as *Aspidoscelis tesselatus* (Say, 1823), with 69 chromosomes (Walker et al. 1997). The 2n = 44 is the most common diploid number in this genus (Carvalho et al. 2015). Therefore, a low diploid number could represent an ancestral condition. All-acrocentric karyotypes with 2n = 52 (28M + 24m) (Lowe et al. 1970) and 2n = 50 (26M + 24m) (Manríquez-Morán et al. 2000) were reported in *Aspidoscelis deppii* from an unknown location and from Yucatan, respectively. Therefore, the two karyotypes differ in the number of macrochromosomes. Concurrently with Lowe et al. (1970), we found a 2n = 52 (28M + 24m) (Fig. 5D) all-acrocentric chromosome complement in our sample from Chiapas. This result is also consistent with phylogenetic relationships, since a diploid complement 2n = 52 (28M + 24m) was found in other two species so far analyzed, *A. guttatus* Wiegmann, 1834 and *A. lineattissimus* (Cope, 1878), which are closely related to *A. deppii* (Lowe et al. 1970; Carvalho et al. 2015).

##### Genus *Holcosus* Cope, 1862

Ten species formerly assigned to the genus *Ameiva* F. Meyer, 1795 have been reassigned to the genus *Holcosus* and reorganized in three species groups (Harvey et al. 2012). Both species analyzed here are included in the same *H. undulatus* species group, which contains a total of six species (Harvey et al. 2012): *H. chaitzami* Stuart, 1942, *H. festivus* (Lichtenstein et von Martens, 1856), *H. leptophrys* (Cope, 1893), *H. niceforoi* (Dunn, 1943), *H. quadrilineatus* (Hallowell, 1860), and *H. undulatus* (Wiegmann, 1834). The genus *Holcosus* has uncertain relationships within Teiidae (Harvey et al. 2012) and has been considered sister to the genus *Cnemidophorus* (Pyron et al. 2013).

###### Holcosus festivus

Taxon classificationAnimaliaSquamataTeiidae

(Lichtenstein et von Martens, 1856)

57DE0A77-BA6E-54F6-977B-312D7FD880C4

####### Distribution.

This species is found in the lowlands of Tabasco and Mexico down to Colombia; it does not enter in the Yucatan Peninsula.

####### Samples.

RCMX223 (female*), RCMX224 (female*), and RCMX233 (female) from Estación Chajul, Selva Lacandona, Montes Azules, Chiapas, Mexico.

####### DNA taxonomy.

The 600-bp PCR-amplified fragments of the MT-ND2 gene were identical in the two specimens (RCMX223 and RCMX233). The BLASTn search showed that this sequence belongs to *Holcosus festivus*, with 99.8% – 100% identity to *H. festivus* (KR058107, Montes Azules) and 96% identity to the other two *H. festivus* samples (KR058105 and KR058106, Costa Rica).

####### Chromosomes.

Here we report the first karyotype description for *H. festivus* (Fig. 5B). We analyzed two female individuals, both with the diploid number 2n = 50. The karyotype is composed of a gradual series of acrocentric chromosomes: 26 macro- and 24 microchromosomes. The largest pair of chromosomes shows a secondary constriction at the distal end (see discussion below under the *H. undulatus* account).

###### Holcosus undulatus

Taxon classificationAnimaliaSquamataTeiidae

(Wiegmann, 1834)

DC745973-6A68-510D-BC36-981770173E98

####### Note.

Meza-Lázaro and Nieto-Montes de Oca (2015), in a molecular phylogenetic study, proposed the elevation of 9 of the 12 *H. undulatus* subspecies to species rank. However, this change has not been widely accepted by other authors. Therefore, we formally use the previous classification, but we also take in account the results of the Meza-Lazaro and Nieto-Montes de Oca (2015) study.

####### Distribution.

The species is distributed along both coasts of Mexico from southern Nayarit to northern Costa Rica Pacific coast) and from southern Tamaulipas to central Nicaragua (Atlantic coast) including the peninsula of Yucatan.

####### Samples.

RCMX77 (female*) from La Sepultura, Chiapas, Mexico.

####### DNA taxonomy.

The MT-ND2 sequence (556-bp) obtained from the individual from Chiapas has a 99% match to two GenBank sequences of *H. undulatus parvus* Barbour et Noble, 1915 (KR058051 and KR058063). According to Meza-Lazaro and Nieto-Montes de Oca (2015), this subspecies, distributed in the Pacific coast region of Southern Mexico and Northern Guatemala, should be elevated to species rank.

####### Chromosomes.

The specimen analyzed shows a 2n = 50 chromosome number (Fig. 5C). The karyotype comprises a gradual series of acrocentric chromosomes (26M + 24m), as previously described in Castiglia et al. (2010) for *H. undulatus* from Chamela, Biological Station (Jalisco). In the genus *Holcosus*, only *H. festivus* (Chiapas, Castiglia et al. 2010) and *H. undulatus* (Jalisco, present data) have been karyotyped. In *Cnemidophorus*, a possible sister group of *Holcosus* (Pyron et al. 2013), 2n = 50 chromosome complement with one biarmed pair has been reported (Carvalho et al. 2015). Different species of *Kentropyx* Spix, 1825 and *Ameiva* show a 2n = 50 all-acrocentric karyotype, similar to the one found in *Holcosus* (Carvalho et al. 2015). Since these genera span the entire phylogenetic tree of Teiidae, we hypothesize that 2n = 50 all-acrocentric karyotype may represent an ancestral condition. However, to reveal more reliable pattern of chromosomal change, an ancestral state analysis combining karyotype and molecular phylogeny should be made (e.g. Castiglia et al. 2013a).

#### Family Dactyloidae

##### Genus *Anolis* Daudin, 1802

*Anolis* (*sensu lato*) is the most speciose genus among the reptiles, with about 380 recognized species that have been all enclosed in a complete molecular phylogenetic tree by Poe et al. (2017). Most of the mainland species belong to the clade *Norops* Wagler, 1830, a large monophyletic assemblage including nearly 170 species (Poe et al. 2017).

The ancestral karyotype of “beta” Anolis (Norops) consists of 2n = 28 or 2n = 30 chromosomes subdivided in 14 macro- and 14 or 16 microchromosomes without evident sex chromosome heteromorphism (Castiglia et al. 2013b). Another frequently observed chromosome complement in this group has 2n = 40 (24M+16m), which is considered to have been derived from the previous complement through fission events on macrochromosomes (Castiglia et al. 2013b). The presence of heteromorphic sex chromosomes has been repeatedly reported in *Norops*. Moreover, it might have occurred independently in different lineages (Castiglia et al. 2013b, Gamble et al. 2014). Among “beta” *Anolis*, heteromorphic XY chromosomes have been reported in eight species (Castiglia et al. 2013b; Giovannotti et al. 2016). Furthermore, a system with two X chromosomes and one Y (X_1_X_1_X_2_X_2_/X_1_X_2_Y) has been reported in *A. biporcatus* (Wiegmann, 1834) (2n = 29 for males and 2n = 30 for females) (De Smet 1981). This multiple sex-chromosome system also occurs also in other *Anolis* species and it is believed to have been the result of a sex-autosome translocation event (Giovannotti et al. 2016; Kichigin et al. 2016).

###### Anolis capito

Taxon classificationAnimaliaSquamataDactyloidae

Peters, 1863

1B49C987-07F8-5979-A9C0-F3C8470037CE

####### Distribution.

*Anolis capito* has been found from Tabasco and northern Chiapas south to Central America on the Atlantic coast, to Costa Rica and Panama, where it is found on both coasts.

####### Samples.

RCMX217 (female*), RCMX218 (female*) from Montes Azules, Chiapas, Mexico. The specimens were collected close to the northern part of species range and morphologically assigned to *Anolis capito*. Based on morphological studies from populations of almost all the species range, there is no evidence of cryptic species in *A. capito* (Köhler et al. 2005).

####### DNA taxonomy.

We obtained a 685-bp MT-ND2 sequence showing 9% genetic divergence respect to an *A. capito* sequence collected in Costa Rica (GenBank AY909744). Such a high genetic divergence spurred us to perform a complete phylogenetic analysis with the MT-ND2 gene of *Anolis* species available in GenBank (not shown). The sequences from our samples cluster with the GenBank *A. capito* sequence, and together were sister to *A. tropidonotus* Peters, 1863. This tree topology has been already reported by Poe et al. (2017). Summarizing, the very high genetic divergence and discrepancies in diploid chromosome numbers (see below) of morphologically similar individuals recognized as *Anolis capito* indicate the possible existence of cryptic taxa. Further, it is worth noting that the specimens described here seem to have shorter limbs than other *A. capito* (O. Flores-Villela personal observation).

####### Chromosomes.

Gorman (1973) described the karyotype of *Anolis capito*, under the name of *Norops capito*, as 2n = 40 (24M + 16m) with no evidence of heteromorphic sex chromosomes, but no details on the shape of the chromosomes were reported. Our specimens have a 2n = 42 chromosome complement, with 24 micro- and 18 microchromosomes, and no evidence of heteromorphic sex chromosomes but no males have been studied (Fig. 6A).

The specimens presently studied show, along with *Anolis nebuloides* Bocourt, 1973, the highest diploid number within the genus *Anolis*. The macrochromosomes include one pair of metacentric, six pairs of submetacentric, and five pairs of subtelocentric/acrocentric chromosomes. The chromosome shape of two pairs of microchromosomes appears to be biarmed. No heteromorphic sex chromosomes are discernible (unfortunately, no males have been analyzed).

The lack of description of chromosome morphology in Gorman’s study (Gorman 1973) did not allow detailed comparison among the 2n = 40 chromosomal complements. Thus, *Anolis capito* occurs within a group of species with 2n = 40 (Castiglia et al. 2013b) and its additional chromosomal pair is probably due to a fission event. It has already been hypothesized that chromosomal fission is a characteristic trait of *Norops* chromosome evolution (Castiglia et al 2013b; Gamble et al. 2014).

###### Anolis lemurinus

Taxon classificationAnimaliaSquamataDactyloidae

Cope, 1861

E6EB7EE3-D6EA-5011-A01C-0105A36A2ABE

####### Distribution.

Occurs on the Atlantic coast from central Veracruz to central Panama, and on the Pacific coast from Costa Rica to central Panama.

####### Samples.

RCMX214 (male*), RCMX225 (male*) Estación Chajul, Selva Lacandona, Montes Azules, Chiapas, Mexico.

####### DNA taxonomy.

BLAST analysis of the 630-bp MT-ND2 gene sequences from both individuals show 99.5% – 100% of identity with a sequence of *A. lemurinus* from Oaxaca (GenBank KT724761).

####### Chromosomes.

**Figure 8.**
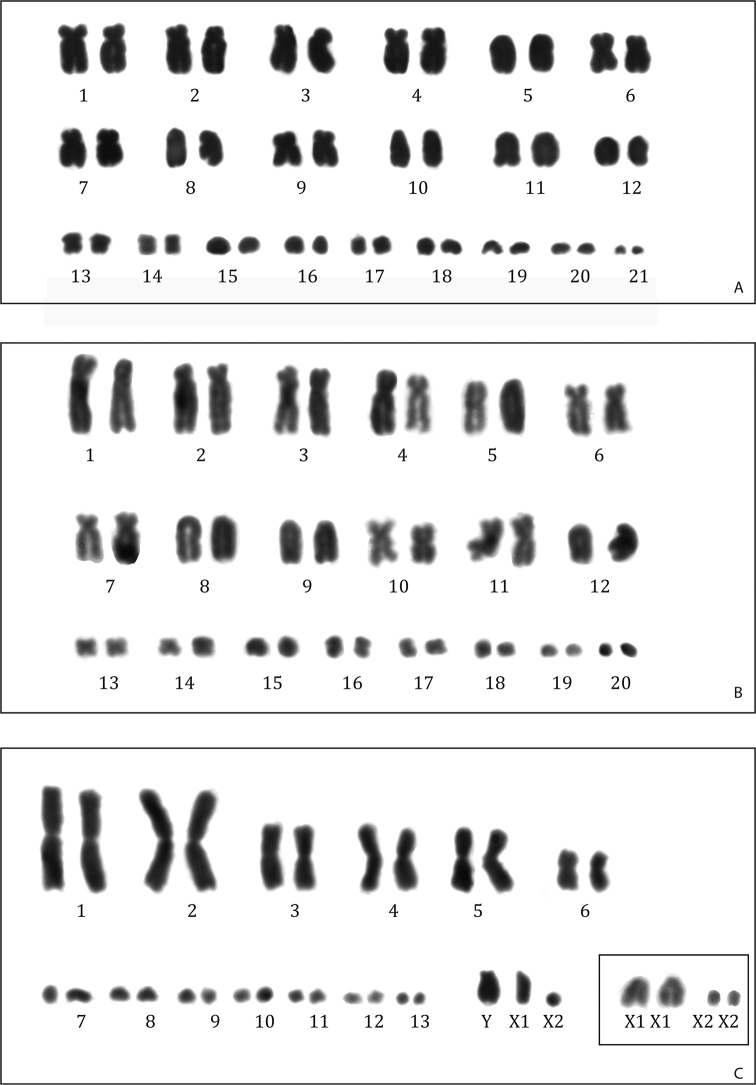
Karyotypes of **A***Anolis capito* (2n = 40, RCMX218 female) **B***Anolis lemurinus* (2n = 40, RCMX214 male) and **C***Anolis uniformis* (2n = 50, RCMX210 male) with YX_1_X_2_ sex chromosomes; in the box the X_1_X_1_X_2_X_2_ (RCMX206 female) sex chromosomes.

No previous chromosomal data are available for *A. lemurinus* and its karyotype is here described for the first time. Both male specimens from Montes Azules have a 2n = 40 (24M + 16m) karyotype (Fig. 8B). The 12 pairs of macrochromosomes include eight pairs of submetacentric and four pairs of subtelocentric/acrocentric chromosomes. The metacentric chromosomes of pair 10 are of different size and may represent heteromorphic sex chromosomes of the XY type.

This karyotype has the same composition in micro- and macrochromosomes as all *Anolis* species with 2n = 40 so far described. Molecular phylogenetics (Poe et al. 2017) place *A. lemurinus* nested within a clade in which all the species so far karyotyped show 2n = 40 (Castiglia et al. 2013b). Ancestral state analysis (Castiglia et al. 2013b) indicates that the 2n = 40 karyotype is derived from by five centric fissions of macrochromosomes from an ancestral 2n = 30. What that should be further investigated are the chromosomal rearrangements occurring within macrochromosomes in the 2n = 40 karyotype.

###### Anolis uniformis

Taxon classificationAnimaliaSquamataDactyloidae

Cope, 1885

3067A92E-261E-5C7F-BDCF-D2F37C86D53F

####### Distribution.

Occurs from southern Tamaulipas to north-central Honduras on the Atlantic coast.

####### Samples.

RCMX201 (male), RCMX203 (male), RCMX205 (male*), RCMX206 (female*), RCMX209 (female), RCMX210 (male*), RCMX215 (male*) and RCMX226 (female*) from Estación Chajul, Selva Lacandona, Montes Azules, Chiapas, Mexico.

####### DNA taxonomy.

The species was formerly included in the *A. humilis* group, but it is now included in the *Draconura* clade (Poe et al. 2017). Over the 780-bp of the MT-ND2 fragment, the GenBank BLAST reports a 99% identity with *A. uniformis* from Belize (KJ954096 and KJ954099).

####### Chromosomes.

We report here the first description of the karyotype of this species (Fig. 8C). The species is characterized by X_1_X_1_X_2_X_2_/X_1_X_2_Y sex chromosome system. In fact, male individuals have a chromosome number 2n = 29 (14M + 15m) and females show 2n = 30 (14M + 16m). The macrochromosomes can be morphologically divided in two pairs of large metacentrics, three pairs of medium sized metacentrics, one pair of small metacentric and one pair of small acrocentric chromosomes. The X_1_ was identified as an acrocentric chromosome and X_2_ as a microchromosome. The Y chromosome is an acrocentric one similar in size to X_1_.

Among the species of the genus *Anolis* with a known karyotype, this species is phylogenetically close to *A. aquaticus* Taylor, 1956 and *A. biporcatus*. Furthermore, *A. biporcatus* has also a similar composition of the sex chromosomes system, even if the morphology of sex chromosomes is different. In fact, the so-called 2n = 30 karyotype is one of the most common karyotypes in *Anolis*. However, three variants of this karyotype, based on the number and shape of macro- and microchromosomes, have been described. Among them, two types of 2n = 29–30 are present, type-A and type-B (Castiglia et al. 2010).

The type-A, typical of *A. biporcatus*, presents a multiple sex chromosomes system where X_1_ is an acrocentric chromosome, X_2_ is a microchromosome, and Y is metacentric similar in size to X_1_.

In our case, the Y is a small acrocentric chromosome, which might have been derived from a pericentric inversion in the submetacentric Y chromosome of the 2n = 29–30 type-A karyotype.Thus, although it is believed that the onset of multiple sex chromosomes in *Anolis* occurs independently (Castiglia et al. 2013b; Gamble et al. 2014), present data suggest that this condition may represent a trait derived from the common ancestor of the two species.

## Conclusions

Combined karyological and DNA taxonomic approaches have allowed us to highlight some interesting taxonomic peculiarities in 10 Mesoamerican lizard species belonging to six genera and five families. The karyotypes of four species, *Phyllodactylus* sp. 3 (*P. tuberculosus* species group), *Holcosus festivus*, *Anolis lemurinus*, and *A. uniformis* are here described for the first time. In *Aspidoscelis deppii* and *Anolis capito*, we found different karyotypes from those previously reported for these species. Moreover, in *A. capito*, the cytogenetic observation is consistent with the considerable genetic divergence at the studied mtDNA marker (MT-ND2), which is indicative of a putative new cryptic species. The anole species here studied exhibited different sex chromosomes configurations including a X_1_X_1_X_2_X_2_/X_1_X_2_Y condition in *A. uniformis* that should be in future studied by molecular cytogenetic techniques.

Another species that may include cryptic taxa is the skink *Scincella cherriei*, for which we found high values of genetic divergence among the specimens from Montes Azules and those from Costa Rica and Nicaragua, comparable to the divergence typical of sister species in skinks. A lower level of genetic divergence, compatible with an intraspecific phylogeographic structure, has been identified for *L. flavimaculatum*. In fact, the studied specimens belong to a mtDNA lineage that is sister with respect to the remaining haplotypes from other populations. However, it should be noted that the novel data represent only the first step in the identification of cryptic species and more efforts are necessary to investigate our assumptions. Both taxonomic revision and the notions related to the chromosome evolution in this hyper-diversified group of reptiles will be worthy of note.

## Supplementary Material

XML Treatment for Scincella assata

XML Treatment for Scincella cherriei

XML Treatment for Phyllodactylus

XML Treatment for Lepidophyma flavimaculatum

XML Treatment for Aspidoscelis deppii

XML Treatment for Holcosus festivus

XML Treatment for Holcosus undulatus

XML Treatment for Anolis capito

XML Treatment for Anolis lemurinus

XML Treatment for Anolis uniformis

## References

[B1] Bauer AM Good DA Branch WR (1997) The taxonomy of the southern African ‘leaf‐toed’ geckos (Squamata: Gekkonidae), with a review of Old World ‘*Phyllodactylus*’ and the description of five new genera. Proceedings of the California Academy of Sciences 49: 447–497.

[B2] Bezy RL (1972) Karyotypic variation and evolution of the lizards in the family Xantusiidae. Contributions in Sciences 227: 1–29.

[B3] Bezy RL (1989) Morphological differentiation in unisexual and bisexual Xantusiid lizards of the genus *Lepidophyma* in Central America. Herpetological Monographs 3: 61–80. 10.2307/1466986

[B4] Bezy RL Camarillo JL (2002) Systematics of xantusiid lizards of the genus *Lepidophyma*. Contributions in Sciences 493: 1–41.

[B5] Bickham JW (1984) Patterns and models of chromosomal evolution in reptiles. In: Sharma AK Sharma A (Eds) Chromosome in Evolution of Eukaryotic Groups (Vol.2). CRC Press, Florida, 13–40.

[B6] Blair C Méndez de La Cruz FR Ngo A Lindell J Lathrop A Murphy RW (2009) Molecular phylogenetics and taxonomy of leaf-toed geckos (Phyllodactylidae: *Phyllodactylus*) inhabiting the peninsula of Baja California. Zootaxa 2027(1): 28–42. 10.11646/zootaxa.2027.1.2

[B7] Blair C Méndez-de la Cruz FR Law C Murphy R (2015) Molecular phylogenetics and species delimitation of leaf-toed geckos (Phyllodactylidae: *Phyllodactylus*) throughout the Mexican tropical dry forest. Molecular Phylogenetics and Evolution 84: 254–265. 10.1016/j.ympev.2015.01.003 25620603

[B8] Carvalho NDM Arias FJ da Silva FA Schneider CH Gross MC (2015) Cytogenetic analyses of five amazon lizard species of the subfamilies Teiinae and Tupinambinae and review of karyotyped diversity the family Teiidae. Comparative Cytogenetics 9(4): 625–644. 10.3897/CompCytogen.v9i4.5371 26753079 PMC4698576

[B9] Castiglia R Annesi F Bezerra AMR García A Flores-Villela O (2010) Cytotaxonomy band DNA taxonomy of lizards (Squamata, Sauria) from a tropical dry forest in the Chamela-Cuixmala Biosphere Reserve on the coast of Jalisco, Mexico. Zootaxa 2508: 1–29. 10.11646/zootaxa.2508.1.1

[B10] Castiglia R Bezerra AMR Flores-Villela O Annesi F Muñoz A Gornung E (2013a) Comparative cytogenetics of two species of ground skinks: *Scincella assata* and *S. cherriei* (Squamata: Scincidae: Lygosominae) from Chiapas, Mexico. Acta Herpetologica 8: 69–73. 10.13128/Acta_Herpetol-11315

[B11] Castiglia R Flores-Villela O Bezerra AMR Muñoz A Gornung E (2013b) Pattern of chromosomal changes in ‘beta’ *Anolis* (*Norops* group) (Squamata: Polychrotidae) depicted by an ancestral state analysis. Zoological Studies 52: 1–60. 10.1186/1810-522X-52-60

[B12] Castiglia R García A Bezerra AMR Flores-Villela O Gornung E (2009) Karyotypic diversification due to Robertsonian rearrangements in *Phyllodactylus lanei* Smith, 1935 (Squamata, Gekkonidae) from Mexico. Rendiconti Lincei 20: 77–82. 10.1007/s12210-009-0005-4

[B13] Clement M Snell Q Walker P Posada D Crandall K (2002) TCS: estimating gene genealogies. Proceedings of the 16^th^ International Parallel and Distributed Processing Symposium 2: e184. 10.1109/IPDPS.2002.1016585 [Accessed 06 October 2020]

[B14] CONANP (2019) CONANP – Comisíon Nacional de Áreas Naturales Protegidas. Áreas Naturales Protegidas. Información espacial de las Áreas Naturales Protegidas, Gobierno de Mexico. http://sig.conanp.gob.mx/website/pagsig/info_shape.htm [Accessed 24 June 2020]

[B15] Darriba D Taboada GL Doallo R Posada D (2012) jModelTest 2: more models, new heuristics and parallel computing. Nature Methods 9: e772. 10.1038/nmeth.2109 PMC4594756 22847109

[B16] De Smet WHO (1981) Description of the orcein stained karyotypes of 27 lizard species (LacertiliaReptilia) belonging to the families Iguanidae, Agamidae, Chamaeleontidae and Gekkonidae (Ascalabota). Acta Zoologica Patholologica Antverpiensia 76: 35–72.

[B17] Deakin JE Ezaz T (2019) Understanding the evolution of reptile chromosomes through applications of combined cytogenetics and genomics approaches. Cytogenetic and Genome Research 157: 7–20. 10.1159/000495974 30645998

[B18] Funk SM Fa JE (2006) Phylogeography of the endemic St. Lucia whiptail lizard *Cnemidophorus vanzoi*: Conservation genetics at the species boundary. Conservation Genetics 7: 651–663. 10.1007/s10592-005-9068-7

[B19] Gamble T Bauer AM Greenbaum E Jackman TR (2008) Out of the blue: a novel, trans‐Atlantic clade of geckos (Gekkota, Squamata). Zoologica Scripta 37(4): 355–366. 10.1111/j.1463-6409.2008.00330.x

[B20] Gamble T Geneva AJ Glor RE Zarkower D (2014) *Anolis* sex chromosomes are derived from a single ancestral pair. Evolution 68(4): 1027–1041. 10.1111/evo.12328 24279795 PMC3975651

[B21] García A Ceballos G (1994) Guía de campo de los reptiles y anfibios de la costa de Jalisco, México. Fundación Ecológica de Cuixmala, AC.

[B22] García de Miranda E Falcón de Gyves Z (1986) Nuevo Atlas Porrúa de la Republica Mexicana. (7a. edición). Porrua, 219 pp.

[B23] García‐Vázquez UO Contreras-Arquieta A Trujano-Ortega M Nieto‐Montes de Oca A (2018a) A new species of *Gerrhonotus* (Squamata: Anguidae) from the Cuatro Ciénegas Basin, Coahuila, Mexico. Herpetologica 74: 269–278. 10.1655/HERPETOLOGICA-D-17-00013

[B24] García‐Vázquez UO Nieto‐Montes de Oca A Bryson Jr RW Schmidt‐Ballardo W Pavón‐Vázquez CJ (2018b) Molecular systematics and historical biogeography of the genus *Gerrhonotus* (Squamata: Anguidae). Journal of Biogeography 45: 1640–1652. 10.1111/jbi.13241

[B25] Giovannotti M Cerioni PN Slimani T Splendiani A Paoletti A Fawzi A Barucchi VC (2017) Cytogenetic Characterization of a population of *Acanthodactylus lineomaculatus* Duméril and Bibron, 1839 (Reptilia, Lacertidae), from Southwestern Morocco and insights into sex chromosome evolution. Cytogenetic and Genome Research 153: 86–95. 10.1159/000484533 29183018

[B26] Giovannotti M Trifonov V Paoletti A (2017) New insights into sex chromosome evolution in anole lizards (Reptilia, Dactyloidae). Chromosoma 126: 245–260. 10.1007/s00412-016-0585-6 27001473

[B27] Gorman GC (1973) The chromosomes of reptiles, a cytotaxonomic interpretation. In: Chiarelli B Capanna E (Eds) Cytotaxonomy and Vertebrate Evolution.Academic Press, London, 349–424.

[B28] Hardy LM Raymond LR Harris S (2017) The karyotype of *Plestiodon anthracinus* (Baird, 1850) (Sauria: Scincidae): a step toward solving an enigma. Southeastern Naturalist 16(3): 326–331. 10.1656/058.016.0318

[B29] Harvey MB Ugueto GN Gutberlet Jr RL (2012) Review of teiid morphology with a revised taxonomy and phylogeny of the Teiidae (Lepidosauria: Squamata). Zootaxa 3459: 1–156. 10.11646/zootaxa.3459.1.1

[B30] HGNC (2019) HUGO Gene Nomenclature Committee. https://www.genenames.org [accessed 07 May 2020]

[B31] Honda M Ota H Kohler G Ineich I Chirio L Chen SL Hikida T (2003) Phylogeny of the lizard subfamily Lygosominae (Reptilia: Scincidae), with special reference to the origin of the New World taxa, Genes and Genetic Systems 78: 71–80. 10.1266/ggs.78.71 12655139

[B32] Johnson JD Mata-Silva V García-Padilla E Wilson LD (2015) The herpetofauna of Chiapas, Mexico: composition, physiographic distribution, and conservation status. Mesoamerican Herpetology 2: 272–329.

[B33] Kichigin IG Giovannotti M Makunin AI Kabilov MR Tupikin AE Barucchi VC Splendiani A Ruggeri P Rens W O’Brien PC (2016) Evolutionary dynamics of *Anolis* sex chromosomes revealed by sequencing of flow sorting-derived microchromosome-specific DNA. Molecular Genetics and Genomics 291(5): 1955–1966. 10.1007/s00438-016-1230-z 27431992

[B34] King M (1981) Chromosome change and speciation in lizards. In: Atchley WR Woodruff DS (Eds) Evolution and Speciation.Cambridge University Press, 262–265.

[B35] Koch C Flecks M Venegas PJ Bialke P Valverde S Roedder D (2016) Applying n-dimensional hypervolumes for species delimitation: unexpected molecular, morphological, and ecological diversity in the Leaf-Toed Gecko *Phyllodactylus reissii* Peters, 1862 (Squamata: Phyllodactylidae) from northern Peru. Zootaxa 4161(1): 41–80. 10.11646/zootaxa.4161.1.2 27615910

[B36] Kocher TD Thomas WK Meyer A Edwards SV Pääbo S Villablanca FX Wilson AC (1989) Dynamics of mitochondrial DNA evolution in animals: amplification and sequencing with conserved primers. Proceedings of the National Academy of Sciences 86(16): 6196–6200. 10.1073/pnas.86.16.6196 PMC297804 2762322

[B37] Köhler G Schulze A Vesely M (2005) Morphological variation in *Norops capito* (Peters, 1863), a wide-spread species in southeastern Mexico and Central America. Salamandra 41: 129–136.

[B38] Lee JC (1996) The Amphibians and Reptiles of the Yucatán Peninsula. Comstock Publishing Associates, Cornell University Press, Ithaca, 500 pp.

[B39] Leigh JW Bryant D (2015) PopART: Full-feature software for haplotype network construction. Methods in Ecology and Evolution 6(9): 1110–1116. 10.1111/2041-210X.12410

[B40] Linkem CW Diesmos AC Brown RM (2011) Molecular systematics of the Philippine forest skinks (Squamata: Scincidae: *Sphenomorphus*): testing morphological hypotheses of interspecific relationships. Zoological Journal of the Linnean Society 163(4): 1217–1243. 10.1111/j.1096-3642.2011.00747.x 32336789 PMC7165859

[B41] Lowe CH Wright JW Cole CJ Bezy RL (1970) Chromosomes and evolution of the species groups of *Cnemidophorus* (Reptilia: Teiidae). Systematic Zoology 19: 128–141. 10.2307/2412450

[B42] Macey JR Schulte II JA Larson A Tuniyev BS Orlov N Papenfuss TJ (1999) Molecular phylogenetics, tRNA evolution, and historical biogeography in anguid lizards and related taxonomic families. Molecular Phylogenetics and Evolution 12(3): 250–272. 10.1006/mpev.1999.0615 10413621

[B43] Manríquez-Morán NL Villagrán-Santa Cruz M Méndez de la Cruz F-R (2000) Origin and evolution of the parthenogenetic lizards, *Cnemidophorus maslini* and *C. cozumelae*. Journal of Herpetology 34: 634–637. 10.2307/1565287

[B44] Matos NB Ferreira M Jesus Silva F Rodrigues MT Santos da Silva E Garcia C (2016) Taxonomy and evolution of *Tropidurus* (Iguania, Tropiduridae) based on chromosomal and DNA barcoding analysis. Journal of Herpetology 50: 316–326. 10.1670/13-221

[B45] Meza-Lázaro RN Nieto-Montes de Oca A (2015) Long forsaken species diversity in the Middle American lizard *Holcosus undulatus* (Teiidae). Zoological Journal of the Linnean Society 175: 189–210. 10.1111/zoj.12264

[B46] Murphy RW Blair C Méndez-de la Cruz FR (2009) A new species of leaf-toed gecko, genus *Phyllodactylus* (Squamata: Gekkota: Phyllodactylidae) from Guerrero, Mexico. South American Journal of Herpetology 4(1): 17–24. 10.2994/057.004.0103

[B47] NALCMS North American land Change Monitoring System (2020) North American land cover. http://www.cec.org/tools-and-resources/north-american-environmental-atlas/map-files [Accessed 24 June 2020]

[B48] Nielsen SV Daza JD Pinto BJ Gamble T (2019) ZZ/ZW sex chromosomes in the endemic Puerto Rican leaf-toed gecko (*Phyllodactylus wirshingi*). Cytogenetic and Genome Research 157(1–2): 89–97. 10.1159/000496379 30685761

[B49] Nielsen SV Pinto BJ Guzmán-Méndez IA Gamble T (2020) First report of sex chromosomes in night lizards (Scincoidea: Xantusiidae). Journal of Heredity 111(3): 307–313. 10.1093/jhered/esaa007 32076711

[B50] Noonan BP Pramuk JB Bezy RL Sinclaire EA de Queiroz K Sites Jr JW (2013) Phylogenetic relationships within the lizard clade Xantusiidae: Using trees and divergence times to address evolutionary questions at multiple levels. Molecular Phylogenetics and Evolution 69: 109–122. 10.1016/j.ympev.2013.05.017 23742886

[B51] Olmo E Signorino GG (2005) Chromorep: a reptiles chromosomes database. http://chromorep.univpm.it/ [accessed 06 October 2020]

[B52] Palacios-Aguilar R Santos-Bibiano R Flores-Villela O (2018) A New Species of *Lepidophyma* (Squamata: Xantusiidae) from the Pacific Lowlands of Guerrero, Mexico. Journal of Herpetology 52(3): 327–331. 10.1670/17-061

[B53] Palumbi SR Martin A Romano S McMillan WO Stice L Grabowski G (1991) The simple fool’s guide to PCR, version 2.0. University of Hawaii, Honolulu, 45 pp.

[B54] Pellegrino KCM Santos RML Rodrigues MT Laguna MM Amaro RC Yonenaga-Yassuda Y (2009) Chromosomal evolution in the Brazilian geckos of the genus *Gymnodactylus* (Squamata, Phyllodactylidae) from the biomes of Cerrado, Caatinga and Atlantic rain forest: evidence of Robertsonian fusion events and supernumerary chromosomes. Cytogenetic and Genome Research 127: 191–203. 10.1159/000295175 20215729

[B55] Poe S Nieto-Montes de Oca A Torres-Carvajal O De Queiroz K Velasco JA Truett B Gray LN Ryan MJ Köhler G Ayala-Varela F Latella I (2017) A phylogenetic, biogeographic, and taxonomic study of all extant species of *Anolis* (Squamata; Iguanidae). Systematic Biology 66(5): 663–697. 10.1093/sysbio/syx029 28334227

[B56] Pokorná MJ Trifonov VA Rens W Ferguson-Smith MA Kratochvíl L (2015) Low rate of interchromosomal rearrangements during old radiation of gekkotan lizards (Squamata: Gekkota). Chromosome Research 23(2): 299–309. 10.1007/s10577-015-9468-6 25665924

[B57] Pyron RA Burbrink FT Wiens JJ (2013) A phylogeny and revised classification of Squamata, including 4161 species of lizards and snakes. BMC Evolutionary Biology 13: 1–93. 10.1186/1471-2148-13-93 23627680 PMC3682911

[B58] QGIS (2017) QGIS 2.18.9 ‘Las Palmas’. Free Software Foundation, Inc., Boston. http://www.qgis.org/en/site/forusers/download.html [Accessed 24 June 2020]

[B59] Rambaut A Drummond AJ Xie D Baele G Suchard MA (2018) Posterior summarization in Bayesian phylogenetics using Tracer 1.7. Systematic Biology 67(5): e901. 10.1093/sysbio/syy032 PMC6101584 29718447

[B60] Ramirez-Bautista A (1994) Manual y claves ilustradas de los anfibios y reptiles de la region de Chamela, Jalisco, Mexico. Cuadernos del Instituto de Biologıa, UNAM, 127 pp.

[B61] Ramírez-Reyes T Flores-Villela O (2018) Taxonomic changes and description of two new species for the *Phyllodactylus lanei* complex (Gekkota: Phyllodactylidae) in Mexico. Zootaxa 4407(2): 151–190. 10.11646/zootaxa.4407.2.1 29690191

[B62] Ramírez-Reyes T Piñero D Flores-Villela O Vázquez-Domínguez E (2017) Molecular systematics, species delimitation and diversification patterns of the *Phyllodactylus lanei* complex (Gekkota: Phyllodactylidae) in Mexico. Molecular Phylogenetics and Evolution 115: 82–94. 10.1016/j.ympev.2017.07.008 28739370

[B63] Reeder TW Cole CJ Dessauer HC (2002) Phylogenetic relationships of whiptail lizards of the genus *Cnemidophorus* (Squamata: Teiidae): a test of monophyly, reevaluation of karyotypic evolution, and review of hybrid origins. American Museum Novitates 3365: 1–61. 10.1206/0003-0082(2002)365<0001:PROWLO>2.0.CO;2

[B64] Ríos-Muñoz CA (2013) ¿Es posible reconocer una unidad biótica entre América del Norte y del Sur? Revista Mexicana de Biodiversidad 84: 1022–1030. 10.7550/rmb.34170

[B65] Ronquist F Huelsenbeck JP (2003) MRBAYES 3: Bayesian phylogenetic inference under mixed models. Bioinformatics 19: 1572–1574. 10.1093/bioinformatics/btg180 12912839

[B66] Rovatsos M Farkačová K Altmanová M Pokorná MJ Kratochvíl L (2019) The rise and fall of differentiated sex chromosomes in geckos. Molecular Ecology 28: 3042–3052. 10.1111/mec.15126 31063656

[B67] Santos RML Pellegrino KCM Rodrigues MT Yonenaga-Yassuda Y (2007) Banding patterns and chromosomal evolution in five species of Neotropical Teiinae lizards (Squamata: Teiidae). Genetica 131: 231–240. 10.1007/s10709-006-9133-2 17206461

[B68] Savage JM (1982) The enigma of the Central America Herpetofauna: dispersals or vicarance? Annals of the Missouri Botanical Garden 69: 464–547. 10.2307/2399082

[B69] Sinclair EA Pramuk JB Bezy RL Crandall KA Sites Jr JW (2010) DNA evidence for non-hybrid origins of parthenogenesis in vertebrates. Evolution 64: 1346–1357. 10.1111/j.1558-5646.2009.00893.x 19922448

[B70] Udvardi M (1984) The IUCN/UNESCO system of biogeographic provinces in relation to the Biosphere Reserves. In: Unesco-UNEP (Ed.) Conservation science and society.Contributions to the First International Biosphere Reserve Congress, Minsk, Byelorussia/USSR. USSR, 16–19.

[B71] Uetz P Freed P Hošek J (2020) The Reptile Database. http://www.reptile-database.org [accessed on May 2020]

[B72] UNESCO (2018) UNESCO’s commitment to biodiversity, Connecting people and nature for an inspiring future. UNESCO, Paris, 52 pp https://unesdoc.unesco.org/ark:/48223/pf0000265200 [accessed 06, October 2020]

[B73] Walker JM Cordes JE Taylor HL (1997) Parthenogenetic *Cnemidophorus tesselatus* complex (Sauria: Teiidae): a neotype for diploid *C. tesselatus* (Say, 1823), redescription of the taxon, and description of a new triploid species. Herpetologica 53: 233–259.

[B74] Weiss AJ Hedges SB (2007) Molecular phylogeny and biogeography of the Antillean geckos *Phyllodactylus wirshingi*, *Tarentola americana*, and *Hemidactylus haitianus* (Reptilia, Squamata). Molecular Phylogenetics and Evolution 45(1): 409–416. 10.1016/j.ympev.2007.01.006 17346992

[B75] Wilson LD Johnson JD (2010) Distributional patterns of the herpetofauna of Mesoamerica, a biodiversity hotspot. Conservation of Mesoamerican Amphibians and Reptiles 30: e235.

